# Virus Protein-Specific Immune Responses in Selective Depletion of Lymphocyte Populations Using Monoclonal Antibodies in Bolivian Squirrel Monkeys (*Saimiri boliviensis boliviensisv*)

**DOI:** 10.1089/vim.2024.0080

**Published:** 2025-01-02

**Authors:** Pramod N. Nehete, Bharti P. Nehete, Sriram Chitta

**Affiliations:** ^1^ Department of Comparative Medicine, The University of Texas MD Anderson Cancer Center, Bastrop, Texas, USA.; ^2^ The University of Texas Graduate School of Biomedical Sciences, Houston, Texas, USA.

**Keywords:** squirrel monkey, rituximab, 7Pt-3F9, CD8, CD20, immune suppression

## Abstract

The increasing use of immune suppressive monoclonal antibodies in the treatment of organ transplant recipients and patients with oncologic, neurological, and autoimmune diseases can lead to serious morbidity and mortality from the reactivation of viral agents that persist in humans. The squirrel monkey polyomaviruses are naturally found in Bolivian squirrel monkeys (SQM) and may be a useful model for the study of polyomavirus-associated pathogenesis and experimental treatment and prevention strategies. Two diverse groups of squirrel monkeys were given, a single dose of an anti-B cell antibody (rituximab) resulting in complete depletion of B cells (CD20+), while an anti-CD8 monoclonal antibody (7 pt-3F9) resulted in a transient depletion of CD8+ lymphocytes compared with control animals (group with no infusion with either of the monoclonal antibodies). The animals remained clinically healthy, with no pathological symptoms suggesting that the intensity and/or duration of immune suppression were inadequate to trigger pathogenic reactivation of the latent polyoma and herpes viruses. We observed a transient reduction in circulating plasma cytokines, IL-2, IFN-γ, and IL-12 reduced JC and BK viral protein-specific proliferative responses in both the CD8 and CD20 depletion groups. This study clearly elucidates the consequences of the use of depletion monoclonal antibodies in immune suppression modalities in the treatment of human malignancies and during transplantation, and SQM acts as a good model in the selection of dosage at which activation of latent viruses is at a minimum, with no pathological consequences.

## Introduction

Polyomaviruses have limited host ranges, a characteristic that makes the development of animal models of human polyomavirus infection difficult. While BK virus (BKV) can induce tumors in hamsters, rodent cells do not support BKV replication (Tognon et al., 2003; Abend et al., 2009). JC virus (JCV) has been shown to induce brain tumors in both owl monkeys and squirrel monkeys after 1–3 years, but neither BKV nor SV40 displayed this oncogenic potential in owl monkeys (Zaragoza et al., 2005; Del Valle et al., 2008). There has been limited study of the innate and adaptive immune responses to BKV infection in humans. Studies by several investigators have documented the pattern of BKV-specific antibody production that is typical of virus infections, with the development of IgM soon after infection followed by the development of IgG antibodies (Saleh et al., 2020; Flaegstad et al., 1988; Randhawa et al., 2006). In one study, a minority of the pregnant women had rising BKV-specific hemagglutination inhibition (HAI) titers and the development of IgM antibodies coincident with BKV reactivation (Khalili et al., 2013; Shah et al., 1980). Recent seroprevalence studies of human polyomavirus infection have sought to discriminate BKV-specific antibodies from BKV-reactive antibodies that may be cross-reactive with SV40. Modern techniques have proven useful in addressing the long-standing debate over the role of SV40 in human cancers (Butel, 2001; Shah, 2007; Poulin and DeCaprio, 2006). Nonetheless, it remains to be determined which host factors may play a role in the pathogenesis of polyomavirus-associated disease in humans.

Understanding the immunological control of persistent virus infections in immune-competent individuals is a complex endeavor, involving a genetically diverse population of hosts and a diverse collection of viruses. Given the ubiquity of viruses such as cytomegalovirus (CMV), Epstein–Barr virus, BKV, JCV, human papillomavirus, and varicella zoster virus in the human population, and their well-known pathogenic potential among immune-compromised patients, there is an increasing need to discover the basis of immunological control so that it can be selectively restored to prevent or alleviate disease when immune suppression is an unfortunate necessity (Kalter and Heberling, 1990; Meléndez et al., 1969). The high cost of treating acute disease in immune-compromised patients and the public health burden, medical costs, and patient morbidity due to the estimated 20% of human cancers caused by persistent viruses add billions of dollars to the cost of health care annually. Understanding the immunological mechanisms that control persistent viruses in immune-competent patients may hold the key to development of new strategies for prevention and treatment of virus-associated cancers (Meléndez et al., 1969; Stern, 2021; Krump and You, 2018; Mortaz et al., 2016). However, the development of therapeutic strategies to combat these opportunistic viruses has been hindered by the lack of a suitable animal model that recapitulates human disease. The presence of endemic squirrel monkey viruses has been known for over 40 years and all primates used in biomedical research are assumed to harbor persistent viruses asymptomatically, including herpes viruses and polyomaviruses (Kalter and Heberling, 1976; Kalter and Heberling, 1990; Ashkenazi and Melnick, 1962; Rogers et al., 2015; Harvey et al., 2023; Marshall, 2000). Squirrel monkeys are an ideal model for the study of endemic virus reactivation, as they harbor four known viruses that are analogues of opportunistic human viruses: the squirrel monkey polyomavirus (SMPyV), the squirrel monkey CMV (SM-CMV), Saimiri herpesvirus-2 (SaHV-2), and Saimiri herpesvirus-3 (SaHV-3) (Abee, 2000; Ashkenazi and Melnick, 1962). Much like their analogous human viruses, these viruses are pathogenic in the squirrel monkey.

The primary objective of this study was to evaluate the efficacy of anti-CD8 and anti-CD20 monoclonal antibodies for selective binding *in vitro* and for selective depletion of lymphocytes *in vivo* and to monitor immunological consequences specific to immune cell depletion.

## Materials and Methods

### Monkeys, care and housing

Eight male juvenile (31–36 months old, weighing ∼700 g) squirrel monkeys (*Saimiri boliviensis boliviensis*) were used in accordance with the *Guide for the Care and Use of Laboratory Animals* (ILAR/NRC, 1996), the U.S. Department of Agriculture through the Animal Welfare Act (7 USC 2131), 1985, and the Animal Welfare Standards incorporated in 9 CFR Part 3, 1991. Monkeys were housed in stainless steel primate cages, with standard size and dimensions in an environmentally controlled space at an ambient temperature of 64–84°F (17–28°C) and relative humidity of 30–70% and continuously monitored by the Johnson Controls, Inc., Metasys System for Extended Architecture (MSEA) v2.2. Cages were cleaned daily and sanitized twice monthly.

### Diet and water

Animals were fed the New World Primate Diet (Purina #5040) in an amount determined by the study veterinarian based on the average body weights. In addition, they were fed either a fresh fruit or vegetable daily. Specialty foods, such as seeds, peanuts, raisins, yogurt, cereals, frozen juice cups, and peanut butter, or contaminants in the diets, were not expected to affect the results of this study. Each animal received a daily enrichment to include treats and visual and auditory stimulation. Water potable for human consumption was provided *ad libitum* through control devices.

#### Justification for the selection of the test system

This test system was selected based on the need for a high degree of homology with humans for selective depletion of lymphocyte subsets, as used clinically in humans. The monoclonal antibodies are analogous to antibodies used in the treatment of human oncologic, rheumatologic, and neurological conditions.

#### Prestudy certification of animal health and acclimation

All study animals were housed in pairs and allowed to acclimate for a minimum of 7 days before the study start date. All monkeys were examined by a veterinarian within 7 days for overall condition, body weight, and body temperature before study initiation. Blood samples were taken from a peripheral vein and analyzed for a complete blood count (0.5–1 mL per animal) on a Siemens Advia 120 Hematology Analyzer. Serum chemistry parameters were analyzed on an Olympus AU400e® Chemistry Immuno Analyzer. Only healthy animals were released for study.

### Test articles

The 7Pt-3F9, a mouse monoclonal antibody that reacts with rhesus and squirrel monkey CD8 alpha chain, and rituximab, which reacts with B cells, were provided by the NIH-funded Nonhuman Primate Reagent Resource at Boston, MA. The monoclonal antibody anti-CD8 alpha [7Pt-3F9] was produced and distributed by the Nonhuman Primate Reagent Resource (NIH Nonhuman Primate Reagent Resource Cat# anti-CD8 alpha [7Pt-3F9], RRID AB-2819280). Rituximab (Genentech, CA) was purchased from the University of Texas MD Anderson Cancer Center pharmacy, Houston, TX.

### Dose preparation and method of administration

The monoclonal antibodies were infused intravenously (IV). Blood **s**amples for CBC (Complete Blood Count), chemistry, and immune analysis, were collected before dosing on day 0 and afterward at days 7, 14, 28, 42, and 56.

### In vivo depletion of T and B cells

Study animals were divided into the following three groups: Group 1 (*n* = 2) were control animals that received saline infusions; Group 2 (*n* = 3) received a single 20 mg/kg intravenous infusion of 7 pt-3F9; and Group 3 (*n* = 3) received a single 50 mg/kg intravenous infusion of rituximab. Beginning a week before the first dose of immune suppression, blood, urine, and fecal specimens were collected for hematological and metabolic analyses. The experimental design is shown in Table 1.

**Table 1.** Experimental Design

Group No.	Route of administration	mAb name	mAb dose	Total volume administered (mL)	No. of animals
1	IV	None	0 mg/kg	0.5–1	2
2	IV	7Pt-3F9	20 mg/kg	0.5–1	3
3	IV	Rituximab	50 mg/kg	0.5–1	3
Total number of animals					8

### Flow cytometry

Phenotypic characterization of lymphocytes in peripheral blood from the monkeys was performed by cell staining and flow cytometric analysis as described previously (Nehete et al., 2013). Briefly, 100 μL of whole blood from each sample was added to each 12 × 75 mm polystyrene test tube (Falcon, Lincoln Park, NJ, USA) containing preadded monoclonal antibodies against CD3, CD4, CD8, CD16, CD20 (CD3-FITC, clone SP-34; CD4-PerCP, clone L200; all from BD Pharmingen (San Diego, USA) and CD8-PE, B5 Invitrogen (Waltham, MA), and incubated for 15 min at room temperature in the dark. Red blood cells were lysed with FACS lysing solution (Becton Dickinson, USA). Cells were washed and resuspended in 2% formaldehyde. A separate tube of 100 µL of blood was stained with monoclonal CD3-FITC, clone SP-34; and anti-CD16-PE (3G8, BD Pharmingen), for 15 min, and red blood cells were lysed as described above. When all the samples were properly stained, cells were acquired on a flow cytometer and analyzed using FlowJo software (Tree Star, Inc., Ashland, OR). Compensation and isotype controls were used to define specificity, acquired on a flow cytometer. The antibody detail is shown in Table 2.

**Table 2.** Antibody Information

Antibody	Color	Clone	Catalog #	Isotype	Company
CD3	APC-Cy7	SP34.2	557757	Mouse IgG1,λ	BD Pharmingen
CD4	PerCP	L200	550631	Mouse IgG1k	BD Pharmingen
CD8	FITC	3B5	MHCD08014	Mouse IgG2ak	Invitrogen
CD16	BV650	3G8	563692	Mouse IgG1k	BD Pharmingen
CD20	APC	L27	340941	Mouse IgG1	BD
Goat anti-mouse IgG	Alexa Fluor 647		115-605-062		Jackson Research Lab
Goat anti-human IgG	FITC		F0132		Sigma Aldrich

### In vitro assay for binding of depletion antibodies in squirrel monkey

One hundred microliters of whole blood were incubated with 1 μg depletion antibodies either 7Pt-3F9 or rituxan for 30 min at room temperature. Excess antibodies were removed by washing with PBS. A conjugated secondary antibody (1 μg) was added for a 30-min incubation followed by RBC (Red Blood Cells) lysis using the BD RBC lysis buffer (1X), washed once with FACS wash buffer, and cells were fixed in 300 μL of 1% paraformaldehyde solution before acquiring on FACS Celesta.

### Cytokine multiplex assays

The Nonhuman Primate Cytokine kit with IL-2, IL-4, IL-6, IL-10, IL-12/23(p40), IFN-γ, and TNF-α was purchased from Millipore Corporation (Billerica, MA). Plasma concentrations of cytokines were determined according to the manufacturer’s protocols. Multianalyte profiling was performed on the Bio-Plex 200 system and the Microplate Platform (Luminex X MAP technology). Calibration microspheres for classification and reporter readings as well as sheath fluid were also purchased from Millipore Corporation. Acquired fluorescence data were analyzed by the Bio-Plex Manager 5.0.

### In vitro stimulation

Blood samples were collected in EDTA from the femoral vein at different time points. Before the separation of peripheral blood mononuclear cells (PBMCs) from the blood samples, plasma was collected and stored immediately at −80°C until analyzed. The PBMCs prepared from the blood samples by the standard Ficoll-Hypaque density-gradient centrifugation were used for various immune assays.

The proliferation of PBMC samples from the monkeys obtained at different time points during the study was determined by the standard [^3^H] thymidine incorporation assay. Aliquots of the PBMC (10^5^/well) were seeded in triplicate wells of 96-well plates and stimulated for 7 days with the Con A (Concanavalin A), LPS (Lipopolysaccharide) and PWM (pokweed mitogen) (each at 5 µg/mL final concentration) and BK polyomavirus VP1 protein and JCV polyomavirus VP1 protein (Abcam Inc., Cambridge, MA) (each at 1 µg/mL final concentration). The culture medium served as negative control. The proliferative response was measured as counts per minute (CPM) and was calculated as an increase in the radioactivity over that of the cells cultured in the medium alone (Nehete et al., 1995).

#### ELISpot assay for detecting antigen-specific IFN-γ-producing cells

Freshly isolated PBMCs, as described above, were stimulated with the BK polyomavirus and JCV polyomavirus VP1 protein antigens to determine the numbers of IFN-*γ*-producing cells by the ELISpot assay using the methodology reported earlier (Nehete et al., 2001). Briefly, aliquots of PBMCs (10^5^/well) were seeded in triplicate wells of 96-well plates (polyvinylidene difluoride backed plates, MAIP S 45, Millipore, Bedford, MA) precoated with the primary IFN-*γ* antibody. After incubation for 36 h at 37°C, the cells were removed, and the wells were thoroughly washed with PBS and developed as per protocol provided by the manufacturer. Purple-colored spots representing individual cells secreting IFN-*γ* were counted by an independent agency (Zellnet Consulting, New Jersey, NJ) using the KS-ELISpot automatic system (Carl Zeiss, Inc., Thornwood, NY) for the quantitative analysis of the number of IFN-*γ* spot forming cells (SFCs) for 10^5^ input PBMCs. Responses were considered positive when the numbers of SFCs with the test antigen were at least five and were five above the background control values from cells cultured in the medium alone or with the negative control peptide.

INF-α ELISA was measured in the plasma of squirrel monkey using the ELISA kit from PBL as described in the protocol, and results were reported as pg/mL.

### Statistical analysis

For statistical analysis, samples were grouped according to the treatment of animals from which samples were obtained. The Student’s *t-*test was used to compare the means between different groups and the one-way analysis of variance was used to compare the means among different age groups. Only differences with a *p* < 0.05 were significant.

## Results

We explored the possibility of using the Bolivian squirrel monkey as a nonhuman primate (NHP) model for the ability of monoclonal antibodies directed against CD20 and CD8 lymphocytes to deplete specific lymphocyte populations *in vivo*. The objectives of this study were to determine the specificity and duration of lymphocyte depletion by intravenous infusion of candidate monoclonal antibodies reactive to squirrel monkey T and B lymphocytes using flow cytometric analysis and consequences of immune cell depletion on cell-mediated immune functions.

### In vitro binding of depletion antibodies to peripheral blood lymphocytes

**FIG. 1.**
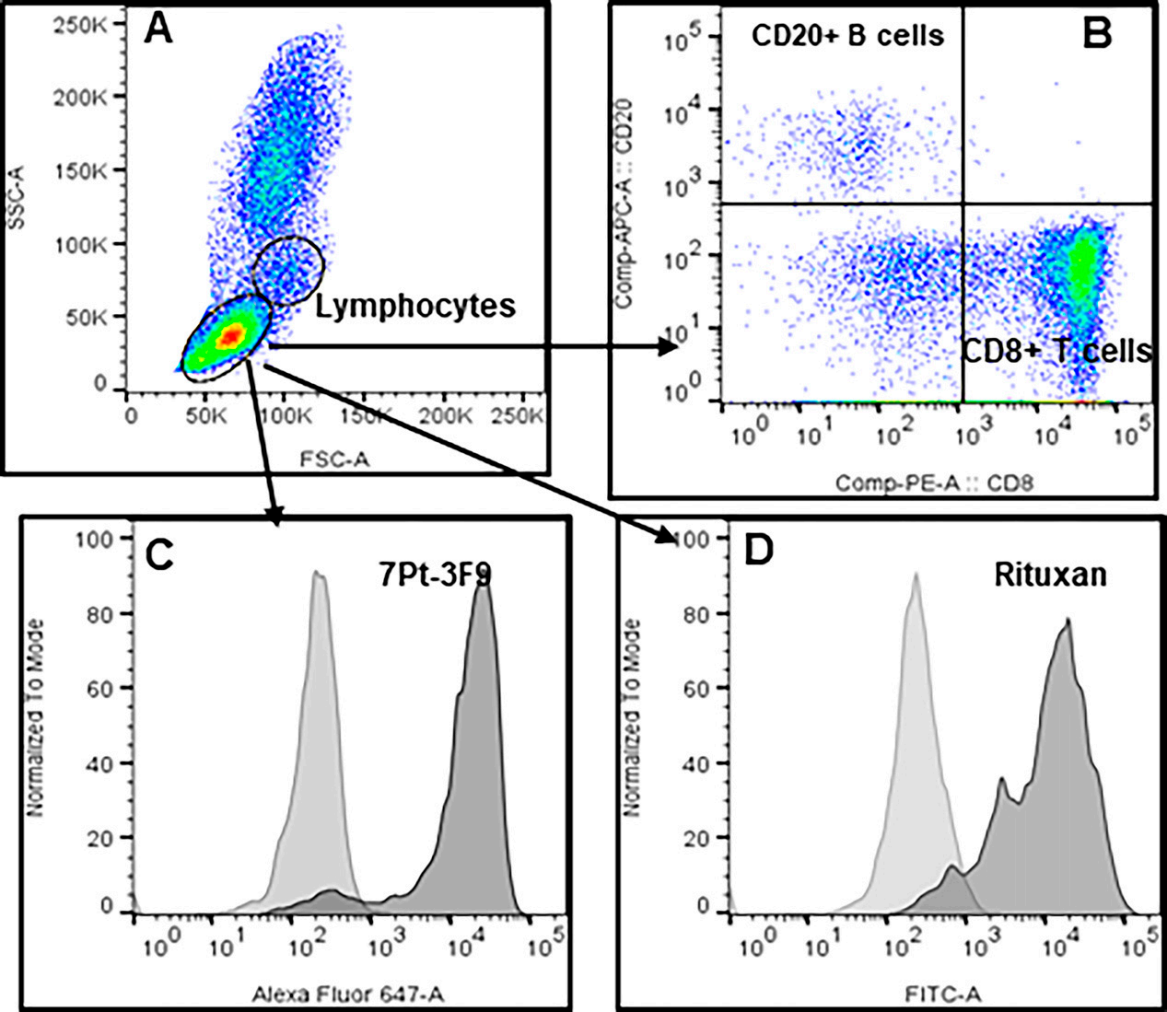
Reactivity of CD8 (clone 7Pt-3F9) and CD20 (rituxan) antibodies used for depletion assessed under *in vitro* conditions using SQM whole blood. Whole blood (100 uL) is incubated with 1 ug antibody for 30 min, excess antibody removed by a single wash, and incubated with a conjugated secondary antibody for another 30 min. Red blood cells (RBCs) were lysed using BD lysis buffer (1×) washed one time with FACS buffer and the pellet of cells fixed in 300 uL 1× paraformaldehyde, to acquire cells using FACS Celesta. Histogram plots **(C** and **D)** show cells incubated with a secondary antibody alone (*light shaded*) and testing antibodies followed by a secondary antibody (*dark shaded*). The dot plots **(A** and **B)** show lymphocyte gating and CD8+ and CD20+ lymphocytes.

Antibodies CD8 (clone 7Pt-3F9) and CD20 (rituximab) used for depletion and were evaluated *in vitro* for binding to squirrel monkey lymphocytes using flow cytometry in whole blood. Results show reactivity with lymphocytes (Fig. 1A–D).

### In vitro analysis of squirrel monkey lymphocyte populations

**FIG. 2.**
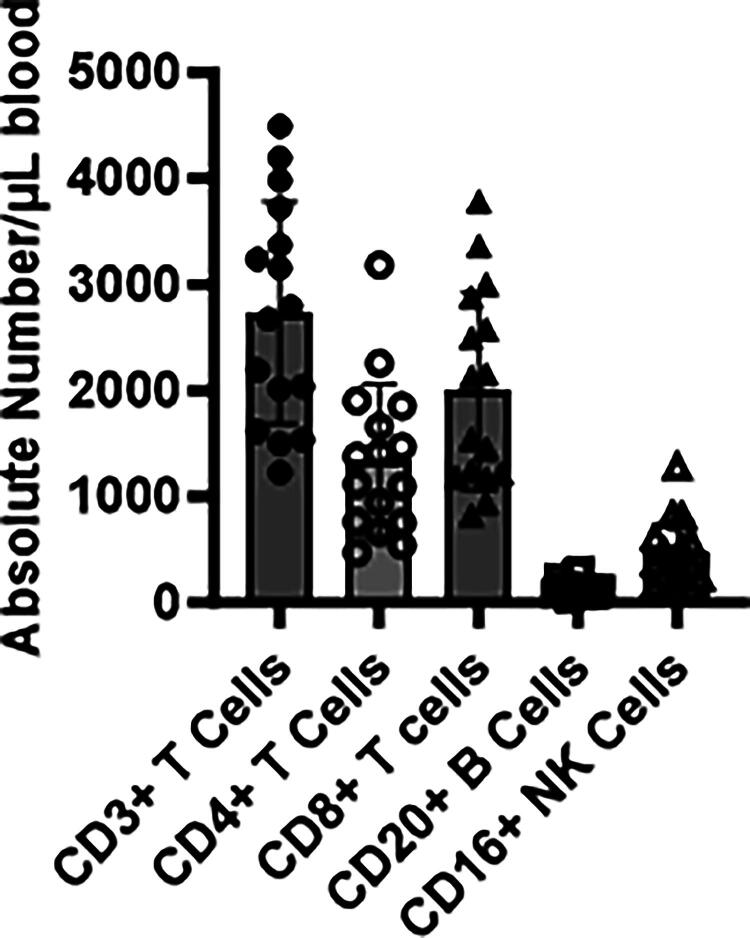
Flow cytometric analysis of whole blood of 52 squirrel monkeys. Absolute numbers of lymphocyte subsets CD3+, CD4+, CD8+, CD20+, and CD16+ are shown.

A series of commercially available human monoclonal antibodies were evaluated for cross-reactivity to Saimiri mononuclear cells using flow cytometric analysis. The clone names, isotypes, and suppliers of the monoclonal antibodies against T and B lymphocyte cell surface markers are shown in Table 1. To establish the normal distribution of the various mononuclear cell subsets such as CD3, CD4, CD8, CD20, and CD16 in the peripheral blood, four-color staining flow cytometry was performed using anti-human antibodies with blood collected from 52 squirrel monkeys. B cells, T cells and their subset cells, and NK cells were determined and summarized as percentages and ranges in Table 3 and as absolute numbers in Figure 2.

**Table 3.** Average Percent of Lymphocyte Subsets in Normal Squirrel Monkeys’ Peripheral Blood

	CD3	CD4	CD8	CD20	CD16
Mean ± SD	81.4 ± 7.4%	40.6 ± 11.5%	55.6 ± 10.4%	7.3 ± 3.3%	11 ± 5.8%
Range	64.5–93.8%	22.1–66.6%	37.5–75.2%	2.8–15.9%	2.8–25%

### In vitro functional immune response analysis of squirrel monkey

We also established *in vitro* assays for the characterization of immune responses to specific antigens and mitogens in SQM. The results shown in Table 4 are summarized from immune responses to common mitogens from 52 animals. These results validate the integrity and reproducibility of the assay when utilizing squirrel monkey lymphocytes and thus were selected for checking immune responses for postdepletion.

**Table 4.** Cytokine Production by Squirrel Monkey Lymphocytes

	PHA	PWM
Proliferation (stimulation index)	11.3 ± 3.8	8.8 ± 4.9
IFN-γ ELISpot	537.4 ± 57.9	330.9 ± 44.1
INF-α ELISA	32 ± 2.8	42.5 ± 3.4

*Proliferation (stimulation index), ELISpot (number of spots/*10^5^ *peripheral blood mononuclear cells or PBMCs), and ELISA (pg/mL) results shown are the average from a study conducted using PBMCs prepared from 52 naive animals*. PHA (Phytohemagglutinin A), PWM (Pokeweed Mitogen).

### In vivo depletion of lymphocyte populations

**FIG. 3.**
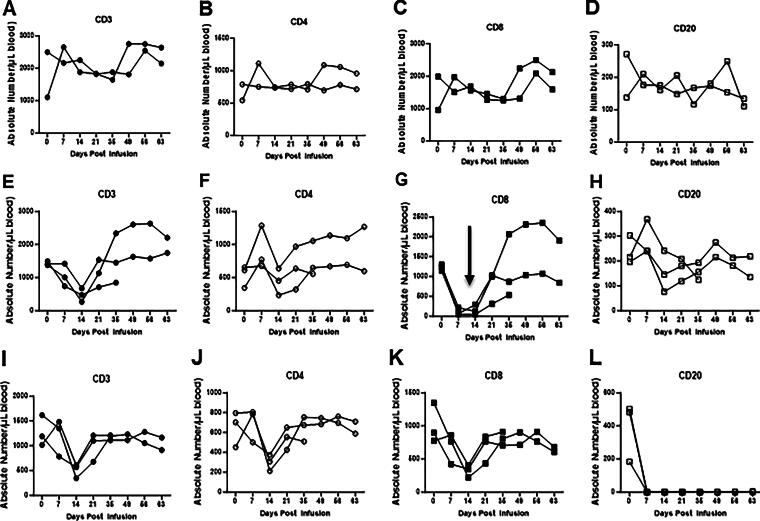
Intravenous infusion of the monoclonal mouse anti-CD8 antibody (7 pt-3F9) or monoclonal mouse/human chimeric anti-human CD20 antibody (rituximab) alone or in saline in the controls in squirrel monkeys. In peripheral blood from squirrel monkeys, expression of absolute numbers of CD3 **(A)**, CD4 **(B)**, CD8 **(C)**, and CD20 **(D)** in control group (*upper panel*). Absolute numbers of CD3 **(E)**, CD4 **(F)**, CD8 **(G)**, and CD20 **(H)** in CD8 depletion group (*middle panel*). Absolute numbers of CD3 **(I)**, CD4 **(J)**, CD8 **(K)**, and CD20 **(L)** in CD20 depletion group (*lower panel*).

The monoclonal antibody7pt-3F9 was administered IV once at 20 mg/kg and rituximab was administered once IV at a dose of 50 mg/kg. The goal of antibody treatments is to sustain 80% reduction in circulating target cells. T and B lymphocytes responses were assessed to specifically characterize the extent of immune suppression and for correlation with the development of clinical disease. Quantitation of lymphocyte subsets CD3 (T cell), CD 4 (helper cell), CD8 (cytotoxic T cells), and CD20 (B cells) was measured by flow cytometry using whole blood samples as shown in Fig.3 A-L). Stained and fixed cells were acquired on a flow cytometer and analyzed using FlowJo software (Tree Star, Inc., Ashland, OR). Compensation and isotype controls were used to define specificity.

**FIG. 4.**
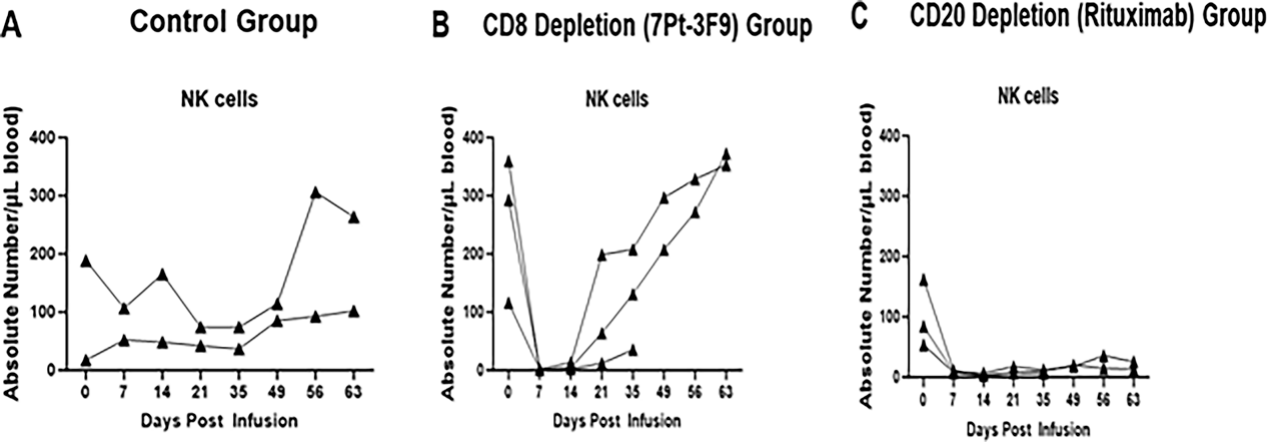
Expression of NK cells in control saline-treated group **(A)**, in monoclonal mouse anti-CD8 antibody (7 pt-3F9) group **(B)**, and in the monoclonal mouse/human chimeric anti-human CD20 antibody (rituximab) group **(C)**.

Expression of NK cells in saline as control group, CD8^+^ lymphocyte and CD20+ lymphocyte depletion in peripheral blood from squirrel monkeys, after intravenous infusion of monoclonal mouse anti-CD8 antibody (7 pt-3F9) or monoclonal mouse/human chimeric anti-human CD20 antibody (rituximab) alone (Fig. 4). Infusion of saline in the controls had no effect on the number of NK cells in peripheral blood from monkeys (Fig. 4A). We observed in the CD8 depletion group, transient depletion of NK cell expression (Fig. 4B) and in the CD20 depletion group, a prolonged depletion of NK cells (Fig. 4C).

Rituximab-induced B cell depletion has a potential effect on T cell function and B cell depletion (Liossis and Sfikakis, 2008). Rituximab is important in understanding various chronic inflammatory responses such as in patients with primary Sjögren’s syndrome (Pollard et al., 2013), autoimmune bullous skin disease (Berkani et al., 2019), and B cell depletion can improve disease in patients with no pathogenic autoantibodies (Berkani et al., 2019; Fillatreau, 2018).

#### Plasma cytokine levels in CD8 (7Pt-3F9)- and CD20 (rituximab)-depleted squirrel monkeys

**FIG. 5.**
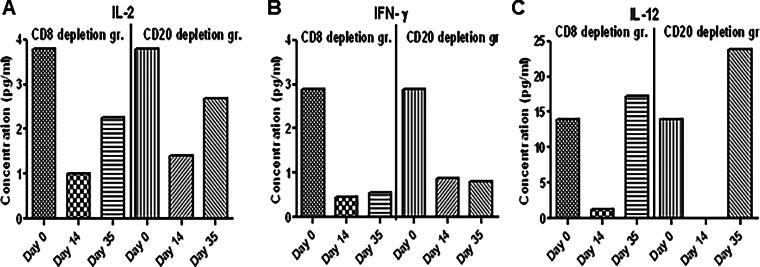
A multiplex nonhuman primate cytokine Plex panel was used to measure analytes in plasma of CD8- and CD20-depleted SQM according to the procedure described in the kit. Acquisition and data were analyzed by the Bio-Plex manager 5.0 (Bio-Rad, Hercules, CA).

Circulating cytokines were measured in plasma collected from the 7 pt-3F9 and rituximab groups using the Multiplex Cytokine Kit (Millipore). We observed a decrease in IL-2 (Fig. 5A) and IFN-γ (Fig. 5B). IL-12 (Fig. 5C) decreased on day 14 but recovered on day 35 in both groups.

#### In vitro T cell proliferative responses to mitogens and proteins from JCV and BKV

**FIG. 6.**
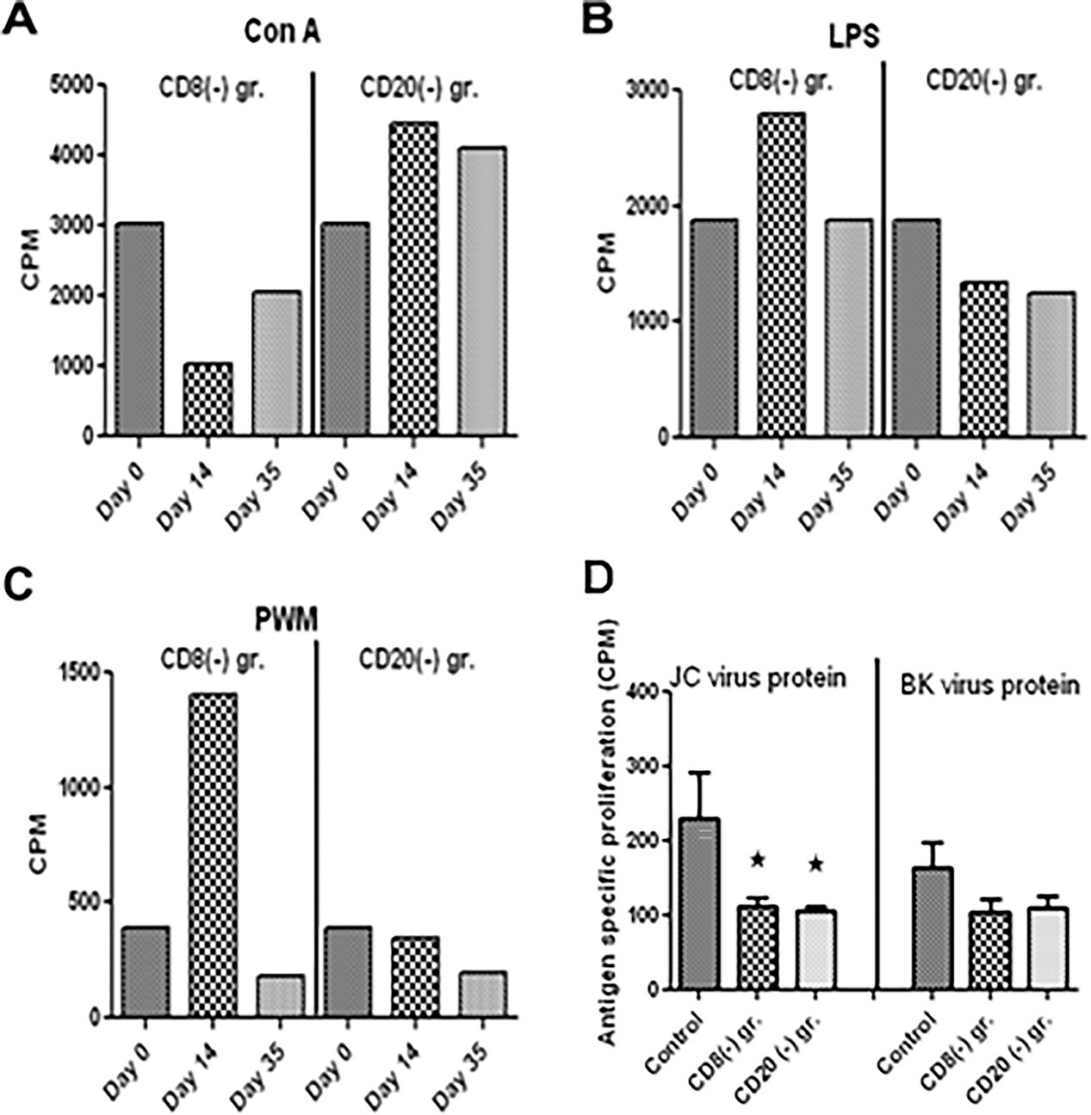
Peripheral blood mononuclear cells (PBMCs) were isolated from blood from controls, CD8 antibody-, and CD20 antibody-treated monkeys, and were *in vitro* stimulated with Con A **(A)**, LPS **(B)**, and PWM **(C)**, JC virus protein **(D)** and BK virus protein **(E)** (each at 1/mL/mL) in triplicate wells of 96-well plates for 6 days. On day 5 of incubation 1uci per well of 3Htritum was added in each well and harvested on a semiautomatic cell harvester (Skatron) and the filter was counted on b-counter. Cell proliferation is expressed as CPM (counts per minute) above medium as background.

The squirrel monkey is a carrier of SMPyV and after depletion of CD8 or CD20 cells, we expect expression of BKV and JCV on cells. Therefore, we isolated PBMCs from the 7 pt-3F9 and rituximab groups, stimulated overnight with Con A (T cell responses), LPS and PWM mitogens (B cell responses), and JC and BK viral proteins for proliferation assay. We observed a transient reduction in Con A-, LPS-, and PWM-specific responses, but no changes in JC and BK protein-specific response. Proliferative responses as CPM above medium as background are shown in Figure 6.

#### In vitro T cell IFN-γ ELISpot responses to mitogens and proteins from BKV and JCV

**FIG. 7.**
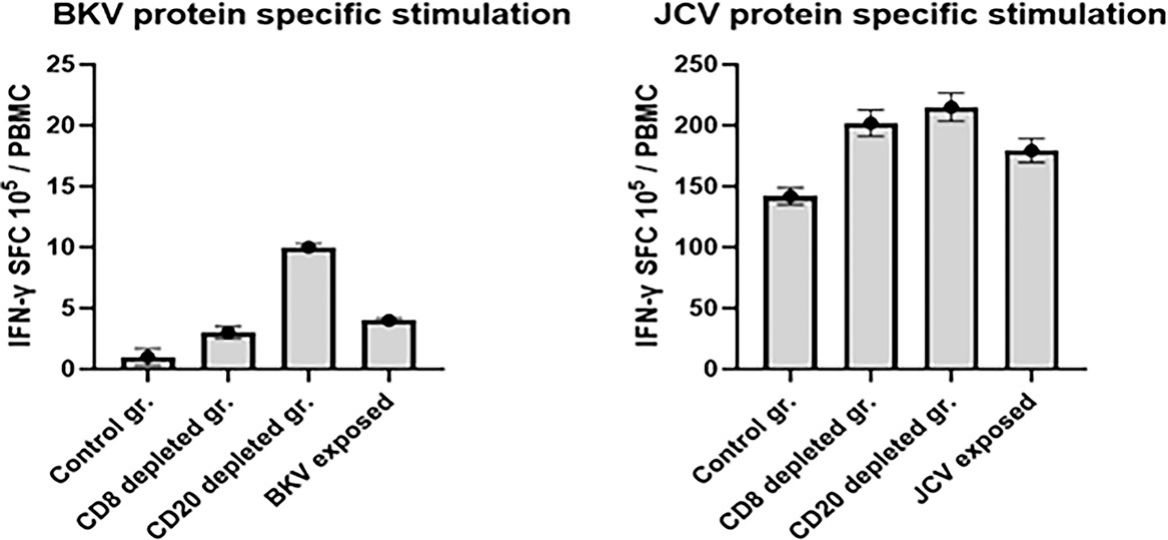
PBMCs (10^5^/well) were incubated with 1 ug/mL of protein of BK and JC virus and seeded in triplicate wells of 96-well plates (polyvinylidene difluoride-backed plates, MAIP S 45, Millipore Bedford, MA), precoated with the primary IFN-*γ* antibody. After incubation for 36 h at 37°C, the cells were removed, and the wells were thoroughly washed with PBS and developed and counted using the KS-ELISpot automatic system (Carl Zeiss, Inc., Thornwood, NY) as per protocol provided by the manufacturer. Responses were considered positive when the numbers of spot-forming cells (SFCs) with the test antigen were at least five above the background control values from cells cultured in the medium alone or with the negative control peptide.

Furthermore, we also measured BKV and JCV protein-specific responses in IFN-γ ELISpot assay. PBMCs from the 7 pt-3F9 and rituximab groups were stimulated for 36 h and developed as described in the Materials and Methods section. Since squirrel monkey is a carrier of polyomavirus (Spy), after depletion of B and CD8 cells, we observed virus-specific IFN-γ responses in the ELISpot assay. The number of IFN-γ SFCs is shown in Figure 7.

## Discussion

BKV and JCV are ubiquitous in the human population and are believed to have evolved to their present state of commensalism over thousands of years. Seroprevalence studies have revealed that BKV infection usually occurs in the first decade of life, with more than 50% of children having BKV antibodies by the time they reach their 5th birthday (Moudgil and Jordan, 2016; Reploeg et al., 2001). Despite this high prevalence of early childhood infection, there is no identifiable disease or clinical syndrome associated with primary BKV infection in immune-competent children (Reploeg et al., 2001; Laskin and Hirsch, 2021). BKV persistence is believed to be lifelong, and the kidney is considered the primary site of persistence in the human body. However, the pathophysiology of BKV infection remains poorly understood and the mode of transmission has not been defined. The anti-human antibodies cross-reactive to PBMCs from squirrel monkeys suggests a phylogenetic conservation and opens new perspectives for vaccinology and immunopathology research in this experimental NHP host.

Potent immunosuppression, especially T lymphocyte depletion, is known to facilitate persistent virus reactivation and predispose patients to opportunistic virus diseases. In the present study, we sought to determine the effect of MAb infusions on the targeted subsets of lymphocytes in peripheral blood and the role of T and B lymphocytes depletion in the squirrel monkey model. First, we demonstrated the *in vitro* cross-reactivity of squirrel monkey lymphocytes of 7 pt-3F9 and rituximab. As expected, squirrel monkey T and B cell lymphocytes were selectively depleted by 7 pt-3F9 and rituximab. Eight juvenile male squirrel monkeys were divided into three groups. Three animals received 7 pt-3F9, an anti-CD8 monoclonal antibody that selectively depletes cytotoxic T lymphocytes, and three animals received rituximab, an anti-CD20 monoclonal antibody that selectively depletes B lymphocytes. Two animals receive placebo infusions of normal saline as the control group.

CD8^+^ T lymphocytes were quantitated by use of a PE-conjugated MAb that was able to bind to CD8 in the presence of 7 pt-3F9, as reported elsewhere (Permar et al., 2004). CD20^+^ B lymphocytes were quantitated by use of a FITC-conjugated anti-CD20 MAb. Flow cytometric analysis using anti-CD20 provided similar results, as reported elsewhere (Permar et al., 2004). When CD8^+^ lymphocytes were undetectable, >95% of the remaining lymphocytes were CD20^+^ or CD4^+^ lymphocytes. Moreover, when CD20^+^ lymphocytes were undetectable, >95% of the remaining lymphocytes were CD3+ lymphocytes.

The total number of CD20+ B cells was effectively suppressed for more than 2 months and lasted longer till end of study. Rituximab is a chimeric monoclonal antibody directed toward CD20, a pan–B cell surface marker that has proved very effective in depleting normal and malignant B lymphocytes *in vivo* and is widely used in the treatment of B cell malignancies, particularly B cell non-Hodgkin’s lymphoma (Safdari et al., 2016). It has been reported that rituximab treatment may decrease peripheral T cells and tissue-resident T cells by abolishing antigen presentation by B cells and enhance regulatory T cells (Liossis and Sfikakis, 2008). In the last 6 years, rituximab has also been used in the treatment of several autoimmune diseases, and the results have been promising. In rheumatoid arthritis (RA), findings of earlier open-label trials suggested that B cell depletion protocols based on rituximab could be an effective therapy in seropositive patients, and this has now been confirmed in a phase II trial (Edwards et al., 2004; Buch et al., 2011; Schioppo and Ingegnoli, 2017). In our experiment, we also observed rituximab induced a profound depletion of all peripheral blood B cell populations in squirrel monkeys. CD20+ B cells in the peripheral blood decreased by a mean of 97% in all monkeys for more than 2 months following rituximab therapy. A small number of T cells and natural killer cells expressed low levels of CD20. The absolute numbers of T cells (CD3+) and subpopulations of T cells (CD4+ and CD8+) were depleted following rituximab therapy and returned to circulation pre-rituximab treatment. NK cells (CD16+ CD3−) also depleted following treatment with both groups. In squirrel monkeys, similar to other NHP species, NK cells characteristically express the CD8 marker (Nehete et al., 2013; Nehete et al., 2021) and depleted during CD8 antibody treatment. B cells with an NK cell-like marker as innate immune cells are reported in NHP (Nonhuman primates)(Zhang et al., 2021; Cogswell et al., 2022). During the period of maximal depletion, a few B cells were detected in the peripheral blood. It is interesting, nevertheless, that repopulation did not occur from these cells. As published in RA patients, repopulation occurred predominantly with naive B cells, indicating that repopulation was dependent on a resumption of the production of naive B cells in the bone marrow (Cambridge et al., 2014; Leandro et al., 2006; Abulayha et al., 2010). Residual B cells did not appear to be able to expand and repopulate the peripheral blood (Leandro, 2013; Kurosaki et al., 2015). We were not able to confirm the repopulation of B cells in squirrel monkeys due to the lack of availability of cross-reactive reagents.

Cellular immunity has been implicated for maintaining successful viral latency in healthy subjects. The rate of recovery from measles virus infection in healthy individuals has been correlated with the strength of their cellular immune response (Jacobsen, 1975; Cernescu et al., 1972). In fact, we and others have recently shown that CD8+ T lymphocytes play a major role in control of SHIV replication and clearance of viremia in rhesus monkeys (Metzner et al., 2000; Manuel et al., 2009; Mueller et al., 2009; Collins et al., 2020).

### Significance

This pilot study provides important scientific data as well as suggesting squirrel monkeys may be a good host for the development of an NHP model of SMPyV disease. The studies of the SMPyV extend the understanding of this novel virus and raise important considerations for undertaking the immune suppression of squirrel monkeys. Finally, the immunological assays available for characterization of the squirrel monkey immune system and its function during immune suppression will provide crucial data for understanding the pathogenic potential of endemic squirrel monkey viruses during immune suppression. These preliminary studies provided assurance that the proposed research yields meaningful results leading to important contributions to the health of patients at risk for opportunistic viral diseases. Overall, with extensive and persistent lymphocyte suppression by administration, a one-time single dose of depletion antibody can be an alternative in renal transplantation. In summary, these proposed studies will advance both the medical and scientific understanding of BKV persistence in immune-competent subjects and provide a solid foundation for understanding the pathogenesis of BKV and other opportunistic viruses threatening our increasing population of immune-compromised patients.
